# Force microscopy of the *Caenorhabditis elegans* embryonic eggshell

**DOI:** 10.1038/s41378-020-0137-3

**Published:** 2020-05-04

**Authors:** Roger Krenger, Jan T. Burri, Thomas Lehnert, Bradley J. Nelson, Martin A. M. Gijs

**Affiliations:** 1 Laboratory of Microsystems, Ecole Polytechnique Fédérale de Lausanne, 1015 Lausanne, Switzerland; 2 Multi-Scale Robotics Laboratory, ETH Zurich, Zürich, 8092 Switzerland

**Keywords:** Engineering, Physics

## Abstract

Assays focusing on emerging biological phenomena in an animal’s life can be performed during embryogenesis. While the embryo of *Caenorhabditis elegans* has been extensively studied, its biomechanical properties are largely unknown. Here, we demonstrate that cellular force microscopy (CFM), a recently developed technique that combines micro-indentation with high resolution force sensing approaching that of atomic force microscopy, can be successfully applied to *C. elegans* embryos. We performed, for the first time, a quantitative study of the mechanical properties of the eggshell of living *C. elegans* embryos and demonstrate the capability of the system to detect alterations of its mechanical parameters and shell defects upon chemical treatments. In addition to investigating natural eggshells, we applied different eggshell treatments, i.e., exposure to sodium hypochlorite and chitinase solutions, respectively, that selectively modified the multilayer eggshell structure, in order to evaluate the impact of the different layers on the mechanical integrity of the embryo. Finite element method simulations based on a simple embryo model were used to extract characteristic eggshell parameters from the experimental micro-indentation force-displacement curves. We found a strong correlation between the severity of the chemical treatment and the rigidity of the shell. Furthermore, our results showed, in contrast to previous assumptions, that short bleach treatments not only selectively remove the outermost vitelline layer of the eggshell, but also significantly degenerate the underlying chitin layer, which is primarily responsible for the mechanical stability of the egg.

## A tough egg to crack

By carefully probing the surface of *Caenorhabiditis elegans* eggs, researchers were able to quantify the physical strength of the various layers shielding the developing embryo. This worm species is a popular laboratory model, and the general structure of its embryonic eggshell has been closely analyzed. Little is known about the shell’s mechanical characteristics, however, and researchers led by Roger Krenger of the EPFL and Jan Burri of the ETH in Switzerland employed a technique called cellular force microscopy (CFM) to address this question. CFM entails precisely prodding biological specimens with a microscale probe and collecting measurements of the resulting force. This allowed them to not only measure the rigidity of the intact egg, but also to assess the structural contributions of individual shell layers by performing measurements after using chemical treatments to remove these various strata.

## Introduction

The nematode *Caenorhabditis elegans* is an important model for biomedical research. The mechanisms of several human diseases, including drug-target interactions, may be studied using this organism since many human disease genes and disease pathways have been conserved between *C. elegans* and humans^1–3^. The *C. elegans* embryo is also an interesting model system to study biological processes at a very early stage, e.g., the formation of functional neuronal networks^4^ and fundamental cellular mechanisms, in particular asymmetric cell division^5^. The development of new therapeutic drug agents can benefit from the use of *C. elegans* as well^6^. The eggshell of its embryo has a direct impact on many early developmental events^7^. This 300–400 nm thick multilayer composite structure provides physical support and protection of the developing organism from external cues, prevents small molecules from permeating it, and blocks polyspermy^8^. For several decades, the eggshell of a nematode was thought to consist of three main layers^9,10^. Recently two additional inner layers have been identified through electron micrographs and by diagnostic biochemical treatments. The first of these layers was referred to as either the embryonic layer, perivitelline space, or extra-embryonic matrix^7,8,11,12^. The innermost layer is called the permeability barrier layer, as it appears to be responsible for preventing small molecules from permeating^12^. Here, we focus on the three main morphologically distinct strata of the eggshell, i.e., an outermost thin vitelline layer (VL), a thicker middle chitin layer (CL), and an underlying chondroitin proteoglycan layer (CPGL)^8^. Each of these layers has a specific function. The VL mediates sperm-oocyte binding, CL provides the structural strength of the eggshell, and the CPGL prevents cytoplasm membrane adhesion to the CL and is required to assemble the inner permeability barrier^12^.

Although one of the main functions of the eggshell is to mechanically protect the embryo, its biomechanical properties are largely unknown. Accurate sensing of changes in its mechanical integrity could offer a new way to identify genes that may up- or downregulate the synthesis of specific shell layers. To date, such biomechanical alterations have only been observed qualitatively by eye, e.g., by the loss of the ovoid egg shape, or by evaluating egg breakage rates while exiting the uterus^8^. In this work, we present an extensive and quantitative micro-indentation study of living *C. elegans* embryos using cellular force microscopy (CFM) to extract the biomechanical properties of the eggshell. The CFM is a microrobotic platform for mechanical stimulation and characterization of living entities of different sizes, ranging from individual cells and tissues to full organs^13,14^. Compared to other mechanical characterization tools used for biological samples, such as atomic force microscopy^15–17^, CFM offers the possibility to apply multi-scale loads in the nN and mN range with significantly higher displacement, i.e., indentation depths in the range of several micrometers with nm-resolution. Previously, CFM has been successfully used for the characterization of the cell wall of growing pollen tubes^18–23^. CFM is a particularly suitable tool for the mechanical characterization of *C. elegans* embryos, as it allows substantial bending and stretching of the hard eggshell with high spatial and force resolution. To demonstrate the versatility of our method, we applied different chemical treatments to the embryos that are known to affect the eggshell multilayer structure. Embryos were exposed to sodium hypochlorite (NaOCl), a chemical used to obtain age-synchronized *C. elegans* worm populations, which selectively removes the VL^8,24,25^, and to chitinase, an enzyme that digests the CL. Our micro-indentation study revealed that these shell-degrading treatments have a drastic effect on the mechanical integrity of the eggshell. We established a biomechanical finite element (FEM) model of the embryo, enabling accurate fitting of the experimental force–displacement curves in order to estimate the Young’s modulus of the eggshell layers under different conditions.

## Results

### CFM micro-indentation protocol

**Fig. 1:**
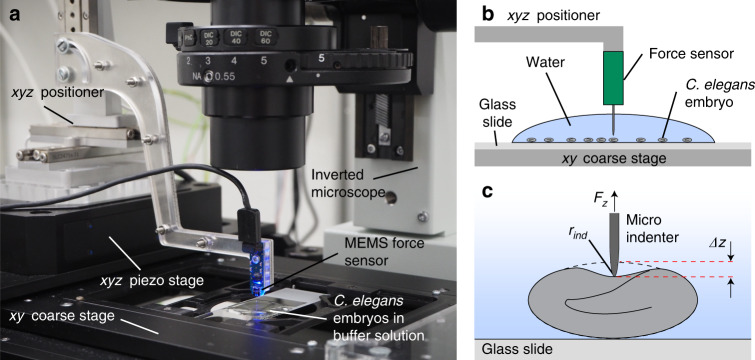
System for micro-indentation measurements on *C. elegans* embryos. **a** Photograph of the cellular force microscope (CFM), integrated with an inverted microscope for high-resolution imaging. The configuration, combining *xyz* precision linear positioners and a piezo system, allows manipulation of the MEMS-based capacitive force sensor with an attached micro-indenter (blue) with nanometer resolution and centimeter travel range. **b** Schematics of the CFM. Once a suitable embryo has been selected by means of the *xy* coarse stage of the inverted microscope, the *xyz* positioner centers the micro-indenter over the egg and moves it in *z* direction close to the shell. **c** Indentation *Δz* of the eggshell is performed by further downward motion of the micro-indenter, controlled via the *xyz* piezo stage. The indenter tip position and the resulting force *F*_*z*_ are recorded simultaneously

Micro-indentation assays were performed by means of a custom CFM setup^20,21^ that was integrated with an optical microscope for high-resolution imaging (Fig. 1a). Nematode embryos in egg buffer were first spotted on a standard microscopy slide, which was inserted into the sample holder of the inverted microscope. After alignment of the sensor tip above the center position and in proximity to the eggshell of a selected embryo, a series of consecutive loading cycles was performed (Fig. 1b). A micro-indentation measurement consists in controlling the indentation depth *Δz* of the sensing microprobe via the *xyz* piezo stage while simultaneously measuring the force *F*_*z*_ generated by the eggshell deformation (Fig. 1c). As a reference for measuring the indentation depth *Δz*, the *z* coordinate of the initial shell contact of the sensor tip during loading, determined at a force threshold level of 0.1 µN, was used. The resulting force–displacement curves *F*_*z*_(*Δz*) were used to extract several mechanical parameters of eggshells subjected to different chemical treatment protocols. Once a preset maximal force *F*_max_ value is reached (loading curve), indentation is stopped and the indenter is automatically retracted. Multiple successive measurements with gradually increasing *F*_max_ values were carried out up to *F*_*z*_^punct^, corresponding to the force level, where irreversible eggshell puncture occurred, typically ranging from ∼5 µN to ∼70 µN depending on the egg treatment. A baseline drift due to viscous friction of water surrounding the sensor tip was taken into account by subtracting a linear fit of the *F*_*z*_(*Δz*) curve during sensor tip approach of the eggshell. Video sequences were recorded during indentation to monitor the morphological changes of the embryos and ImageJ was used for quantification of the parameters.

### Morphological changes upon indentation for different eggshell treatments

**Fig. 2:**
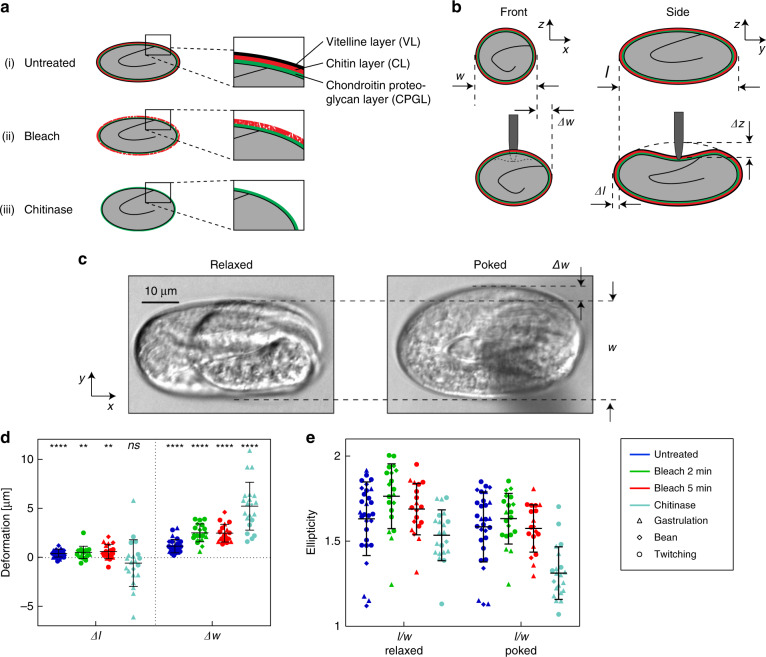
Morphological changes of *C. elegans* embryos upon indentation for different eggshell treatments. **a** Schematic cross-sectional views of the eggshell in its untreated and degraded forms. The untreated eggshell has a trilaminar structure, consisting of an outer vitelline layer, a middle chitin layer and an inner chondroitin proteoglycan layer (ai). Treatment with sodium hypochlorite solution (bleach) selectively removes the VL, but may also attack the underlying CL (aii). Chitinase treatment is applied after bleach treatment to strip away the CL from the embryo shell (aiii). **b** Frontal and lateral schematic cross-sections of an embryo, illustrating the shape changes in width *Δw* and length *Δl* upon indentation with a depth *Δz*. **c** Bright field microscopy images in relaxed and poked states of a live, bleach-treated embryo in the twitching stage. A lateral extension *Δw* upon indentation with respect to the relaxed egg width *w* is well visible (*F*_*z*_ = 5 µN). **d** Scatter plot of embryo elongation *Δl* and widening *Δw* as derived from microscopy images upon indentation. Widening is more prominent than the elongation changes for all eggs and conditions. Paired *t*-test was applied to calculate *p* between *l* and *l* + *Δl* and *w* & *w* + *Δw*, respectively (**p ≤ 0.01; ****p ≤ 0.0001; ns not significant). Force values are *F*_*z*_ = 5.0 µN for untreated and bleached eggs and *F*_*z*_ = 0.5 µN for chitinase-treated eggs. **e** Scatter plot of the embryo ellipticity (ratio *l/w*) in relaxed and poked states for treated and untreated conditions. Very soft chitinase-treated eggs tend to shorten slightly and show stronger widening, but also larger deviation from mean values than untreated and bleached eggs

We performed indentation assays on untreated embryos and embryos exposed to three different conditions (2 min bleach without/with subsequent chitinase treatment, and 5 min bleach, respectively). The chemical treatments affect the structure and mechanical properties of the eggshell membrane, as is schematically indicated in Fig. 2a. The untreated eggshell comprises the full trilaminar structure, consisting of the VL, CL, and CPGL. According to the literature, treatment with bleach solution selectively removes the VL^8^. However, our results show that this treatment also attacks and degrades the CL even after a short incubation time (see Discussion). Chitinase treatment is applied after a bleach treatment to strip the CL away from the embryo. Furthermore, measurements were carried out using eggs at different embryonic development stages, i.e., in the gastrulation, bean, and twitching stages, respectively. The gastrulation stage corresponds to the first stage of freshly laid embryos (∼2.5 to ∼6 h after fertilization), followed by the bean stage (∼6 h to ∼9 h after fertilization), and the twitching stage prior to hatching, during which the embryo starts moving (∼9 h to ∼13 h after fertilization)^26^.

**Fig. 3:**
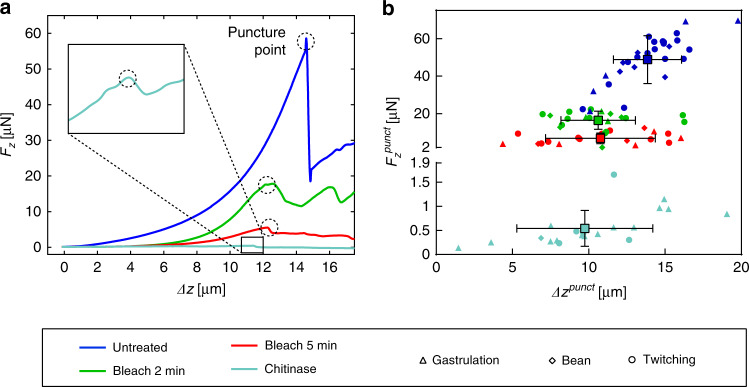
Force-displacement measurements of *C. elegans* embryos. **a** Representative micro-indentation loading curves (after baseline correction), i.e. measured force response *F*_*z*_ of the eggshell vs sensor tip indentation depth *Δz*, for untreated and treated eggs, including displacements beyond eggshell rupture. *F*_*z*_ increases in a nonlinear way with respect to *Δz*, indicating increasing an effective stiffness of the eggshell up to the puncture point. Force response to loading is strongest for untreated eggs (dark blue) and becomes very weak for bleach + chitinase-treated eggshells (light blue), where complete removal of the vitelline layer and the chitin layer is expected. Shell puncture events are marked as dotted circles in the plots. **b** The critical force value *F*_*z*_^punct^ where eggshell puncture occurs is plotted vs the corresponding indentation depth *Δz*^punct^, for different treatments and for all embryonic stages (gastrulation, bean and twitching stage). *F*_*z*_^punct^ decreases drastically with the severity of the treatment. Very low values in the range of 0.5 μN are found after bleach + chitinase treatment, where only the innermost chondroitin proteoglycan layer of the eggshell remains. Untreated eggs can sustain the highest indentation depth *Δz*^punct^, whereas the limit of eggshell deformation is lower but comparable after different treatments

In a first approach, we experimentally quantified the deformation of the spheroid-shaped embryos during indentation by analysis of microscopy bright field images for all conditions. For increasing indentation depth *Δz*, eggs may elongate by *Δl* and widen by *Δw* with respect to the initial unloaded shape as is schematically illustrated in Fig. 2b (length *l* and width *w*, typically ≈50 µm and ≈35 µm, respectively). Figure 2c shows representative high-resolution bright field images of a bleach-treated embryo in the twitching stage, in the relaxed and in a poked state with increased egg width. The embryos, which can be observed through the transparent eggshell, exhibit normal motility after treatment and during indentation. Values for *Δl* and *Δw* were determined at a preset indentation force value *F*_*z*_ = 5.0 µN for untreated and bleach-treated samples. Scatter plots for all conditions are presented in Fig. 2d. A small elongation *Δl* = 0.3 ± 0.4 µm along the major axis of the spheroid was found for untreated eggs, which was only slightly higher for bleach-treated eggshells. As expected, widening *Δw* of the prolate spheroid was more pronounced upon indentation, i.e., *Δw* = 1.1 ± 0.7 µm for untreated samples and about twice that value for bleached embryos. The duration of bleach treatment did not affect this parameter. Nevertheless, elongation and widening of the embryos was always small compared to the indentation depth (*Δz* ≈ 10–15 µm). Chitinase-treated shells are significantly less resistant to mechanical stress and eggshell puncture occurred already at *F*_*z*_ ≤ 1 µN (see Fig. 3b), thus *Δl* and *Δw* values for these samples had to be evaluated at a one order of magnitude lower force value (*F*_*z*_ = 0.5 µN). No measurable deformation was observed for untreated or bleach-treated eggs in this case. Furthermore, we calculated the ellipticity of the individual samples (ratio *l/w*) in relaxed and poked conditions. The results are displayed in Fig. 2e for all conditions. Mean *l/w* values remain in the range between 1.6 and 1.8 for untreated and bleached conditions. Interestingly, chitinase-treated eggs tend to shorten, i.e., to adopt a more spherical shape (*Δl* = −0.6 ± 2.4 µm), even if the variation of the measured values is large. Conversely, the widening *Δw* of chitinase-treated eggs was stronger than for untreated samples (*Δw* = 5.2 ± 2.4 µm). In the relaxed condition, chitinase-treated eggs are more circular than untreated or bleached eggs (*l/w* = 1.5), and in the poked condition, eggs become even rounder (*l/w* = 1.3). Figure 2d, e also provide information about the measurements performed at different embryonic development stages, however, calculated mean values did not show a significant dependence on embryonic stages. A video sequence illustrating the morphological changes of the 4 treated conditions was added to the electronic supplementary information (Vid. S1). Detailed values are summarized in Table S1.

### Experimental indentation curve analysis

Figure 3a shows a set of representative force-indentation curves *F*_*z*_*(Δz)* for untreated, as well as for bleach- and chitinase-treated embryos, respectively. A video sequence in the electronic supplementary information (Vid. S2) illustrates the indentation experiments and shows the shell puncturing. *F*_*z*_*(Δz)* curves for all conditions showed a nonlinear force response, indicating a general trend of increasing effective eggshell stiffness *k(Δz)* for stronger indentation. As *k(Δz)*, corresponding to the slope of *F*_*z*_*(Δz)* at a point *Δz*, is a parameter that is directly extracted from the experimental curve, its value is determined not only by the egg sample properties, but also by the entire indenter system. Figure 3a also illustrates that the mechanical resistance of an embryo with respect to puncture strongly weakens for increasingly shell-degrading treatments. Shell puncture events, corresponding to an abrupt drop in the *F*_*z*_*(Δz)* curves, are marked as black dotted circles. The shell puncture force *F*_*z*_^punct^ was defined as the maximum force *F*_*z*_ that the shell could sustain before rupture. For stronger eggshell treatments *F*_*z*_*(Δz)* curves become flatter, i.e.*, k(Δz)*, as well as the effective stiffness at the shell puncture point *k(F*_*z*_^punct^*)*, decreased. *k(F*_*z*_^punct^*)* values were extracted from the experimental data for all conditions and larval stages and are indicated in Tab. S1. After the sharp drop of *F*_*z*_ at the shell puncture point, the curves proceed at non-zero force levels, most likely due to residual friction forces of the shell and the embryo body on the indenter tip. Notably, the slope of the *F*_*z*_*(Δz)* curves for chitinase-treated eggs is much smaller compared to the other treated and untreated eggs, indicating that the shell itself is generating only a very weak resistance to the indenter in this case and that most of the chitin which provides the mechanical stability has been dissolved. Figure 3b summarizes experimental *F*_*z*_^punct^ values and the corresponding tip indentation depth *Δz*^punct^, i.e., the maximal depth by which the sensor tip may deform the eggshell before irreversible rupture occurred. Data points for the different treatments and embryonic stages are shown. The scatter plot indicates that *F*_*z*_^punct^ mean values clearly diminished with increasing eggshell degradation, i.e., by a factor of ∼2.9 after bleach treatment for 2 min and by a factor of ∼7.2 after bleach treatment for 5 min with respect to untreated shells (*F*_*z*_^punct^ = 49 ± 13 µN). Chitinase treatment after 2 min bleach resulted in a substantial decay of the puncture force, i.e., by a factor of ∼98, compared to untreated embryos. The critical tip indentation depth *Δz*^punct^ was 14 ± 3 µm for untreated eggs and somewhat less after bleach treatment (11 ± 4 µm and 10 ± 4 µm after treatment for 2 min or 5 min, respectively). Chitinase exposure did only slightly affect this parameter with respect to bleach treatment (*Δz*^punct^ = 10 ± 4 µm). To deduce potential mechanical changes of the eggshell in different embryonic stages, mean values were calculated for each stage. We did not, however, observe a clear variation of the shell puncture resistance with respect to the larval development stage. Detailed results for these parameters are presented in Table S1.

### Model for FEM simulations of embryo indentation

To extract the mechanical properties of the embryo eggshell itself from experimental *F*_*z*_*(Δz)* measurements, parameters such as shape and thickness of the shell, as well as the micro-indenter tip size and shape, must be considered. The complexity of the problem makes analytical calculations difficult. Hence, we applied the finite element method (FEM) to model *C. elegans* egg indentation and to calculate *F*_*z*_*(Δz)* loading curves numerically. For this purpose, we model the elastic force component related to membrane deformation and the force generated by the internal egg pressure build-up *p*_int_ upon indentation. Comparing simulated and experimental data allowed the Young’s moduli *E*_shell_ of the eggshells to be determined for different conditions.

**Fig. 4:**
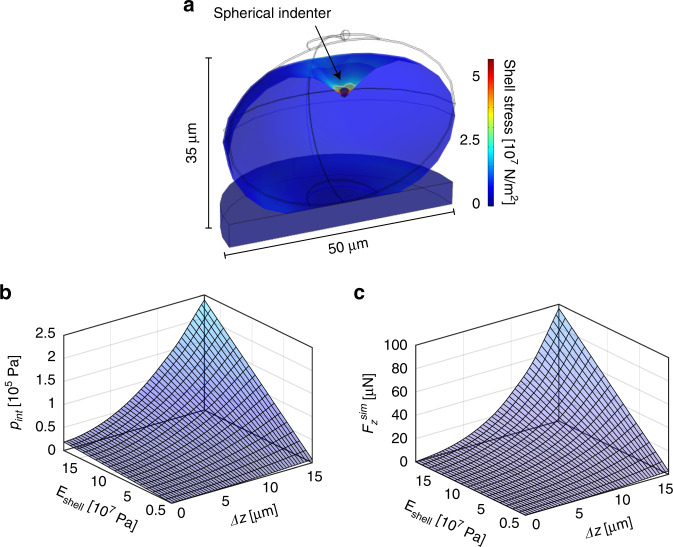
FEM modeling of *C. elegans* embryo indentation experiments. **a** A spheroidal, fluid-filled elastic shell is used as model for indentation simulations and data fitting. Eggshell deformation and resulting membrane stress upon indentation by a spherical indenter are shown for *Δz* = 15 µm (tip radius *r*_ind_ = 1 µm, shell thickness *t* = 300 nm, shell modulus *E*_shell_ = 0.12 GPa, *p*_int_ = 1.6 × 10^5^ Pa). The initial embryo shape in the relaxed state is indicated by gray lines (egg length *l* = 50 µm, width *w* = 35 µm). **b** 3-D representation of the simulated *p*_int_-*Δz-E*_shell_ parameter space. *p*_int,_ the internal egg pressure that builds as a function of the indentation depth *Δz*, is an important parameter required for accurate fitting of the experimental *F*_*z*_*(Δz)* curves. **c** Simulated and interpolated *F*_*z*_^sim^-*Δz-E*_shell_ parameter space. The total force *F*_*z*_^sim^ exerted on the micro-indenter by the eggshell comprises an elastic membrane component and the force related to the internal egg pressure *p*_int_

Our simulations are based on an isotropic linear elastic material model, which is in general a good assumption for small material deformations. This approach requires only two independent eggshell material parameters, i.e., *E*_shell_ and the Poisson ratio *ν*_shell_, to relate stress *σ*_*ij*_ and strain *ε*_*ij*_ components in three dimensions (*i*, *j* = *x*, *y*, *z*) (see SI Eq. 1). We assume a value of *ν*_shell_ = 0.3, which is a generally accepted compromise for biomaterials with largely undefined material properties, but which can be considered as neither fully compressible (*ν* = 0) nor perfectly incompressible (*ν* = 0.5). The eggshell geometry was approximated as a prolate spheroid, which coincides well with the actual *C. elegans* egg shape. The shell thickness was taken as *t* = 300 nm, based on TEM images from Olsen et al.^12^, using uniform material parameters. The egg is considered to be filled with an incompressible fluid, *i.e*. the internal egg volume is considered constant during indentation. A spherical tungsten indenter with tip radius *r*_ind_ = 1 µm presses against the embryo from the top, which lies on a glass baseplate. Standard material parameters of tungsten and glass were used for the rigid components of the model setup. A representative example of the numerical model applied to the embryo is shown in Fig. 4a. The computed stress that builds up in the deformed shell is displayed by a color scheme, assuming an indentation depth *Δz* = 15 µm (i.e., the largest value considered for the simulations), a representative shell modulus *E*_shell_ = 0.12 GPa and *p*_int_ = 1.6 × 10^5^ Pa. The highest stress arises directly under the indenter tip, corresponding to the shell rupture point for *F*_*z*_ > *F*_*z*_^punct^. Stress in the membrane is negligible at locations more than a few micrometers away from the indentation point.

### Determination of the egg shell Young’s moduli

In order to extract the Young’s modulus *E*_shell_ of the *C. elegans* embryonic eggshell for a specific condition from the experimental indentation curves, first the internal egg pressure *p*_int_ that builds as a function of the indentation depth *Δz* was derived by means of our model. *p*_int_ not only deforms the spheroidal egg along the minor and major axes but is also an important parameter for accurate fitting of the experimental *F*_*z*_*(Δz)* curves, as both, pressure and elastic membrane forces, contribute to the measured indentation force. The total simulated force *F*_*z*_^sim^ exerted on the micro-indenter by the eggshell was then calculated by integrating the membrane stress components in *z* direction, under consideration of the internal pressure force. A parametric sweep for *E*_shell_ values from 5.0 × 10^6^ Pa to 1.8 × 10^8^ Pa (incremented by *∆E* = 1.0 × 10^7^ Pa) was used in the simulations to cover the relevant *p*_int_-*Δz-E*_shell_ and *F*_*z*_^sim^-*Δz-E*_shell_ spaces. The interior pressure surface *p*_int_(*Δz, E*_shell_), shown in Fig. 4b, as well as the simulated indentation force surface *F*_*z*_^sim^(*Δz, E*_shell_), plotted in Fig. 4c, were obtained by interpolation using a cubic spline algorithm. The simulated deformations *Δl*^sim^ *and Δw*^sim^ are shown in Fig. S1.

**Fig. 5:**
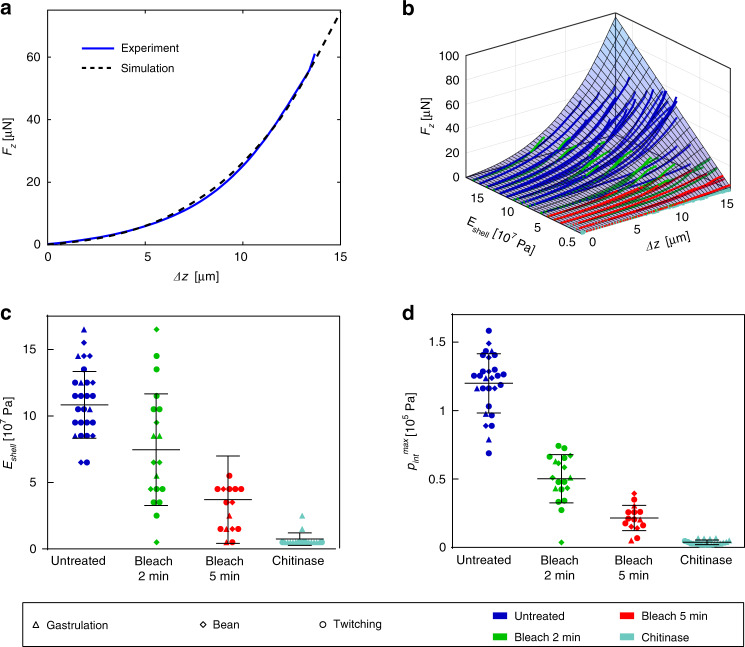
Evaluation of specific mechanical properties of *C. elegans* embryos. **a** Representative force-indentation measurement *F*_*z*_(*Δz*) of an untreated embryo and the best-fitting simulated *F*_*z*_^sim^(*Δz*) curve. Good agreement of both curves is achieved by using a value of *E*_shell_ = 0.12 GPa for the eggshell modulus. **b** The full set of experimental *F*_*z*_(*Δz*) loading curves (solid lines) for all treatments and embryo development stages is superposed to the simulated *F*_*z*_^sim^-*Δz-E*_shell_ parameter space. *F*_*z*_(*Δz*) curves, displayed up to the puncture point, are arranged with respect to the corresponding *E*_shell_ value derived from the best-fitting simulated curve. **c** *E*_shell_ values extracted from the experimental data by applying the described model for all embryonic stages, separated with respect to the corresponding eggshell treatment. The mean value of the Young’s modulus of the shell decreases significantly after bleaching and becomes extremely low after additional chitinase exposure. **d** Maximum values *p*^max^_int_ of the internal egg pressure just before shell rupture for all treatments and embryonic stages

The quality of our model can be evaluated by means of Fig. 5a, where a direct comparison of a representative experimental indentation loading curve for an untreated embryo, and the corresponding best-fitting computed *F*_*z*_^sim^(*Δz*) curve (*E*_shell_ = 0.12 GPa) is plotted. The model reproduces well the nonlinear force response to indentation, and excellent congruence of the two curves over the full *Δz-*range up to the puncture point at *Δz* ≈ 13 µm can be observed. In order to determine the *E*_shell_ value corresponding to a particular experimental *F*_*z*_(*Δz*) curve, the best-fitting simulated curve *F*_*z*_^sim^(*Δz, E*_shell_) was identified by minimizing the sum of squares of the force value difference of a simulated trace and the experimental data $\left( {SS = {\sum} {\left( {F_z^{{\mathrm{sim}}} - F_z} \right)^2} } \right)$, considering the full set of simulated *F*_*z*_^sim^ curves. This protocol was performed for all recorded indentation curves. The results are displayed in Fig. 5b, where the entire set of experimental curves for all conditions and embryo stages is superposed to the simulated and interpolated indentation force surface *F*_*z*_^sim^(*Δz, E*_shell_). Here, the *F*_*z*_(*Δz*) curves are arranged according to the corresponding extracted *E*_shell_ values based on our model assumptions (incremented by ∆*E* = 1.0 × 10^7^ Pa). The scatter plot in Fig. 5c groups all extracted *E*_shell_ values for the different eggshell treatments. Deterioration of the shell mechanical properties, i.e., decreasing mean *E*_shell_ values, depending on type and duration of the treatment, can be clearly observed. For untreated samples, a value *E*_shell_ = 0.12 ± 0.03 GPa was found, which decreased considerably, i.e., by a factor of ∼1.3, ∼2.4 and ∼17.2 for 2 min, 5 min bleach and chitinase-treated samples, respectively. Furthermore, we analyzed the maximal internal pressure *p*^max^_int_ characterizing the pressure value just before shell rupture, which could be extracted from the *p*_int_*-Δz-E*_shell_ space (Fig. 4b) by means of the corresponding maximum indentation depth *Δz*^punct^ (Figs. 3b or 4b) and *E*_shell_ values. Since treated shells have lower mean *E*_shell_ and *Δz*^punct^ values, resulting *p*_int_^max^ values are lower in treated cases. These results are plotted in Fig. 5d for the four eggshell treatment conditions. For untreated embryos a value *p*^max^_int_ = 1.2 × 10^5^ Pa was found, whereas *p*^max^_int_ decreased by a factor of ∼2.4, ∼5.2 and ∼32 for 2 min or 5 min bleach-treated, and chitinase-treated samples, respectively. Mean values for *E*_shell_ and *p*^max^_int_ parameters have also been determined for the different embryo developmental stages, but no significant stage dependence was found. Details are summarized in Table S1.

## Discussion

Although there are various studies looking at the biomechanical properties of adult *C. elegans* worms^27–29^, the embryonic stage of this nematode has not been mechanically characterized. Here, we took advantage of the versatility of an integrated CFM-optical microscope setup for measuring the mechanical properties of embryonic eggshells by means of micro-indentation. The system does not require complicated sample preparation protocols and manipulations. Since the sensor tip can operate in a liquid environment, live embryos can be readily probed in a non-invasive manner under physiological conditions by simply locating an egg suspension on a glass slide. We demonstrated that our system enables detecting impairment of the embryo eggshells under various (bio-)chemical conditions. To extract mechanical shell parameters from experimental force–displacement curves *F*_*z*_(*Δz*), a FEM model was used, where the egg in its unloaded state is considered as a non-pressurized ellipsoidal capsule, undergoing an increasing internal pressure build-up during indentation. Available analytical solutions for ellipsoidal shell structures are not easily applicable for our assays, as these solutions address only the non-pressurized or highly pressurized state without taking into account possible variations of the internal pressure as a function of an external parameter, such as the indentation depth *Δz*^30,31^. Furthermore, the pressure increases during the indentation (since the volume inside the egg stays constant), which affects the measured force and adds another layer of complexity. However, very good agreement of experimental and simulated indentation curves was observed in Fig. 5a and revealed that the simple egg model developed in this work was sufficient to reliably extract fundamental biomechanical properties of the C. *elegans* embryo. Local buckling effects, as for example measured during the indentation of thick-walled axisymmetric domes, were not observed during the indentation of *C. elegans* eggs^32^. Our sample is a thin-walled (*t* = 300 nm, *w* = 35 µm) shell structure and filled with an incompressible liquid, which with a pressure increase during the indentation stabilizes the overall convex structure of the egg. The experimental force–displacement curves recorded during embryo indentation experiments show a gradual increase of the force response until shell puncture. The shell puncture event can be optically detected by a decrease in egg circumferences, indicating a pressure drop in the shell, and worm death (Vid. S2).

Force response of the shell to loading was strongest for untreated eggs and became weak for bleach + chitinase-treated eggshells. It is generally assumed that the relatively thick intermediate CL mainly determines the mechanical properties and stability of the eggshell trilayer structure^8,33^. For example, we found a value of *E*_shell_ = 0.12 GPa for the Young’s modulus of the untreated embryonic eggshell. Interestingly, this value falls into the range of the Young’s moduli reported for chitin-protein networks measured in the beak of the squid *Dosidicus gigas*, i.e., 0.03–5 GPa, depending on hydration and protein content^34^. Reported values for pure chitin structures, such as crystalline chitin in dry state (*E* = 40 GPa)^35^ or chitin nanofibers (estimated *E* = 150 GPa)^36^, however, are up to three orders of magnitude higher than the *E*_shell_ value in the present case. For the time being, nothing is known about the exact structure and composition of the CL, nevertheless, our results indicate that chitin in the *C. elegans* eggshell is probably present in a mixture with proteins.

Moreover, we applied NaOCl (bleach) for selectively removing the outermost VL, and chitinase, which dissolves the underlying CL, and measured the impact of these treatments on the mechanical integrity of the shell. Since the CL provides the mechanical stability to the egg, bleach treatments should not significantly modify the recorded *F*_*z*_*(Δz)* curves, and thereby the mechanical shell parameters, with respect to untreated embryos. On the contrary, our experiments revealed a clear effect of bleach treatment. Even a short treatment drastically changed the mechanical integrity of the embryonic egg, resulting in lower resistance to puncture or lower Young’s moduli *E*_shell_ compared to untreated embryos. Furthermore, as shown qualitatively in Fig. 3a and quantitatively in Fig. 3b and Fig. 5c, the impact increased for longer incubation times (i.e., 2 min vs 5 min). Our observations indicate that the CL is partially removed or at least weakened under these conditions, a fact that disagrees with previous claims in literature, assuming that NaOCl only attacks the VL^8,24,25^. Subsequent treatment with chitinase is expected to completely remove the CL structural backbone of the eggshell, which can indeed be concluded from our measurements, showing extremely low Young’s moduli and puncture forces in this case.

Staining of the CL in *C. elegans* embryos at different developmental stages, using either immunogold-labeled chitin-binding domain probe or wheat germ agglutinin labeling, indicated that the CL undergoes modifications during embryo development, but it is unclear how exactly the CL changes^37,38^. In our study, we performed extensive measurements at all embryo development stages, nonetheless, we could not determine a significant variation of the mechanical properties for a given treatment. These findings indicate that the biomolecular modifications occurring in the shell during ex-uterus embryogenesis of wild-type *C. elegans* do not affect its mechanical properties. Nevertheless, the capability of the CFM to detect mechanical alterations of the eggshell structure with high sensitivity possibly might be applied in the future to identify genes that are responsible for up- and downregulating layer synthesis in the shell stratum during embryogenesis. Eggshell synthesis begins at fertilization through modifications of the extracellular matrix surrounding the oocyte and proceeds sequentially starting from the outer VL. Interestingly, over 100 genes have been identified by RNA interference, whose suppression generates phenotypes showing an osmotic integrity defect (OID)^39–41^. Thus, a depletion of genes playing a crucial role in the formation of the successive eggshell layers eventually may lead to the disruption of the inner permeability barrier. Nevertheless, the specific function of most of these genes is largely unknown. Some exceptions are the *chs-1* gene, which is likely to be the responsible for chitin synthesis in the CL^37^, *cbd-1*, encoding a component of the VL, and *cpg-1*, synthesizing chondroitin proteoglycan for the CPGL^40^. Furthermore, Carvalho *et al*. identified 310 more gene candidates using bioinformatics that might play a role in eggshell synthesis with 20 of them showing the OID phenotype^6^. Even, if most of these genes are not related to the OID phenotype, they could still have a notable impact on eggshell synthesis. Tools that enable assessing the significance of such genes based on other phenotypic criteria are therefore of interest, especially the micro-indentation technique presented in this paper that accurately probes the biomechanical integrity of the eggshell.

## Conclusion

In this work, we conducted a quantitative study of the mechanical properties of the *C. elegans* eggshell. The assays were performed by micro-indentation measurements, taking advantage of a custom CFM setup that was integrated with an optical microscope for high-resolution imaging of the embryos during indentation. We performed micro-indentation assays of *C. elegans* embryos in an untreated state and in three modified conditions, i.e., exposure to two different bleach treatments with subsequent chitinase exposure. Application of these protocols was expected to selectively remove the outermost VL and the underlying CL of the eggshell. We concluded from our experiments, however, that the CL is also affected by the bleach treatment. Experimental micro-indentation curves revealed that the eggshell elastic force *F*_*z*_ increased in a nonlinear way as a function of the indentation depth *Δz* for all four conditions. Mechanical parameters, specifically the Young’s modulus *E*_shell_ of the shell, were extracted from experimental data by means of a simple numerical ellipsoid-indentation model. Our results clearly indicate that the mechanical integrity of the eggshell is strongly affected by the applied chemical treatments, especially after removal of the CL by successive bleach/chitinase exposure. With our study, we demonstrate that CFM is an accurate and versatile tool to determine the mechanical properties of natural *C. elegans* eggshells, as well as of chemically modified shells. This approach opens the way for more advanced embryo assays in view of correlating mechanical properties with other phenotypic or genetic parameters.

## Materials and methods

### Age-synchronized *C. elegans* culture and embryo harvest

*C. elegans* N2 wild-type worms were obtained from the Caenorhabditis Genetics Center (CGC). Standard nematode growth medium (NGM) agar plates were provided by the EPFL Solution Preparation Facility (EPFL SV-IN). *Escherichia coli* OP50 and S Basal media were prepared following standard protocols^42^. An *E. coli* OP50 bacterial lawn was added to the center of the NGM agar plates, on which worm populations were subsequently grown at room temperature^43^. Age-synchronized worm populations were obtained by a worm bleaching protocol (adapted from Stiernagle et al., described in Krenger et al.)^42,44^, followed by seeding of 500–1000 isolated eggs on fresh NGM plates. The plates were then incubated for a duration of ∼64 h at room temperature until gravid adult worms were obtained. Freshly laid eggs were then harvested by scraping the surface of the agar plate with a cell scraper (Sarstedt, Germany) and collecting the sample in an Eppendorf tube. Eggs were separated from adult worms by sedimentation for 30 s and subsequent removal of the egg-containing supernatant.

### Eggshell treatment protocols

Eggs were suspended in egg buffer (118 mM NaCl, 48 mM KCl, 2 mM CaCl_2_, 2 mM MgCl_2_, 25 mM HEPES, pH 7.3) before applying shell treatments^45^. Untreated eggs were taken directly from this suspension. Bleach treatment was performed in a 0.27 M NaOH + 2% NaOCl solution (bleach) for 2 or 5 min, respectively. Egg samples were then washed three times by repeated centrifugation (1680 rcf for 60 s) and resuspended in egg buffer by vortexing. Chitinase-treated shells were obtained by suspending 2-min bleach-treated eggs in egg buffer containing 5 mg mL^−1^ chitinase from *Streptomyces griseus* (Sigma-Aldrich, Buchs, Switzerland) until the start of the experiments (i.e., for ∼15 min).

### Cellular force microscopy

The system, including the CFM used for micro-indentation, was shown in Fig. 1a. One of the main features of the CFM is a commercially available MEMS-based capacitive force sensor (FT-S100; FemtoTools AG, Buchs, Switzerland) to which the micro-indenter is attached, consisting of a tungsten wire with a shaft diameter of 22 μm and a tip radius *r*_ind_ ≤ 1 μm (T-4-22; Picoprobe R by GGB Industries INC, Naples, FL, USA). A microscopy image of the micro-indenter is shown in Fig. S2. The force sensor encompasses a measurement range of ± 100 µN with a resolution of 15 nN, recording at a sampling rate of 500 Hz. The working principle of the force sensor is described in detail by Sun et al.^46^. The force sensor is fixed to the PMMA arm of an *xyz* positioner with a travel range of several cm and a closed-loop resolution of 50 nm (a stack of three SLC-2475-S; SmarAct, Oldenburg, Germany). This *xyz* positioner is used to place the sensor probe in close proximity to the *C. elegans* embryos prior to the indentation experiment. Fine control of micro-indentation is performed by means of a piezo flexure-guided nanomanipulation system (P-563.3CD PIMars; Physik Instrumente GmbH & Co, Karlsruhe, Germany). The piezo stage allows for continuous and high-precision control of the indenter tip position with a spatial closed-loop resolution of 2 nm in *xyz* direction and a full travel range of 300 µm. The CFM is mounted on an inverted microscope (IX71; Olympus K.K., Tokyo, Japan) using a ×20 objective lens (LUCPlanFL N ×20; Olympus K.K., Tokyo) and a CCD camera (Orca-D2; Hamamatsu Photonics K.K., Hamamatsu, Japan) for high-resolution bright field imaging of the embryo samples. The microscope features a motorized *xy* stage system (M-687 PILine XY; PI GmbH & Co.) with a large travel range of 100 mm × 75 mm and 0.1 µm resolution. Data acquisition and control was implemented in LabVIEW^TM^ and executed using a real-time computer with an integrated field programmable gate array (FPGA) (NI cRIO 9024; National Instruments Corporation (NI), Austin, TX, USA). An analog output module (NI-9022; NI) powers all three axes of the CFM piezo stage, and two analog input modules (NI 9215; NI) monitor the *xyz* positions as well as the force signal. The accuracy of the CFM was previously tested using a Si-traceable force-standard (FS-C 15, Si-METRICS, Limbach-Oberfrohna, Germany) that was calibrated and certified by the Physikalisch-Technische Bundesanstalt (PTB), the National Metrology Institute of Germany^20,21^.

## Supplementary information

Supplementary Information

Supplementary video S1

Supplementary video S2
