# Supplementary material for: Force microscopy of the Caenorhabditis elegans embryonic eggshell
Source: Microsyst Nanoeng. 2020 May 4;6:29. doi: 10.1038/s41378-020-0137-3 (PMC7196560; doi:10.1038/s41378-020-0137-3)
Supplement: Supplementary file 1 — Supplementary Information [file 41378_2020_137_MOESM1_ESM.pdf]

Supplementary Information

FEM simulation of the *C. elegans* eggshell: Hooke's law for isotropic materials in compliance matrix form

Linear elasticity theory for isotropic materials was applied to the components of the *C. elegans* egg model presented in Fig. 4a and the corresponding section in the main article. Hereby, the stress-strain relationship, depending only on two constants, *i.e.* the shell elastic modulus E_{shell} and the shell Poisson ratio ν_{shell} can be written as:

$$\begin{bmatrix} \epsilon_{xx} \\ \epsilon_{yy} \\ \epsilon_{zz} \\ 2\epsilon_{yz} \\ 2\epsilon_{xz} \\ 2\epsilon_{xy} \end{bmatrix} = \frac{1}{E_{shell}} \begin{bmatrix} 1 & -\nu_{shell} & -\nu_{shell} & 0 & 0 & 0 \\ -\nu_{shell} & 1 & -\nu_{shell} & 0 & 0 & 0 \\ -\nu_{shell} & -\nu_{shell} & 1 & 0 & 0 & 0 \\ 0 & 0 & 0 & 2(1+\nu_{shell}) & 0 & 0 \\ 0 & 0 & 0 & 0 & 2(1+\nu_{shell}) & 0 \\ 0 & 0 & 0 & 0 & 0 & 2(1+\nu_{shell}) \end{bmatrix} \begin{bmatrix} \sigma_{xx} \\ \sigma_{yy} \\ \sigma_{zz} \\ \sigma_{yz} \\ \sigma_{xz} \\ \sigma_{xy} \end{bmatrix},$$

where ε_{ij} are the strain and the σ_{ij} stress components (with Cartesian co-ordinates *i, j = x, y, z*), respectively (Eqn. S1).

FEM simulation of the *C. elegans* eggshell: Simulated deformation of the eggshell

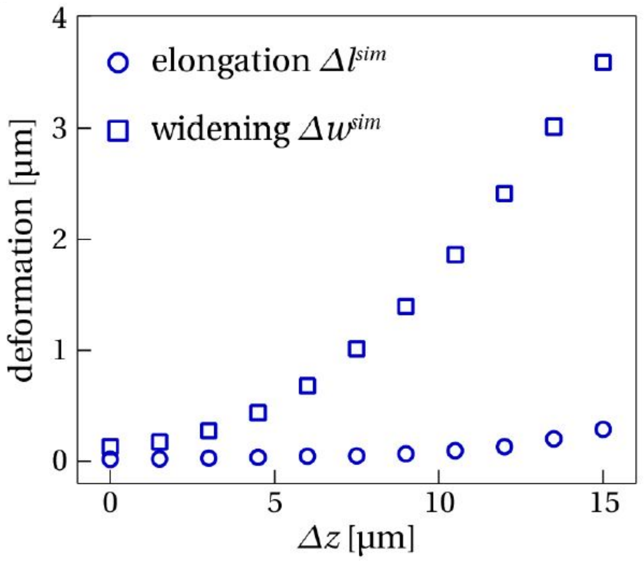

Fig. S1: Simulated elongation and widening of the eggshell upon indentation as a function of the indentation depth (tip radius r_{ind} = 1 µm, shell thickness *t* = 300 nm, shell modulus E_{shell} = 0.12 GPa, p_{int} = 1.6×10^5 Pa).

Micro-indenter geometry

Tungsten wires from Picoprobe R by GGB Industries INC were used as micro-indenter for all experiments. The wires have a shaft diameter of 35 µm and then parabolic narrow down at the tip. The radius at the tip is 1 µm (T-4-22) as given by the manufacturer. Pictures captured with an inverted microscope are shown in Fig. S1.

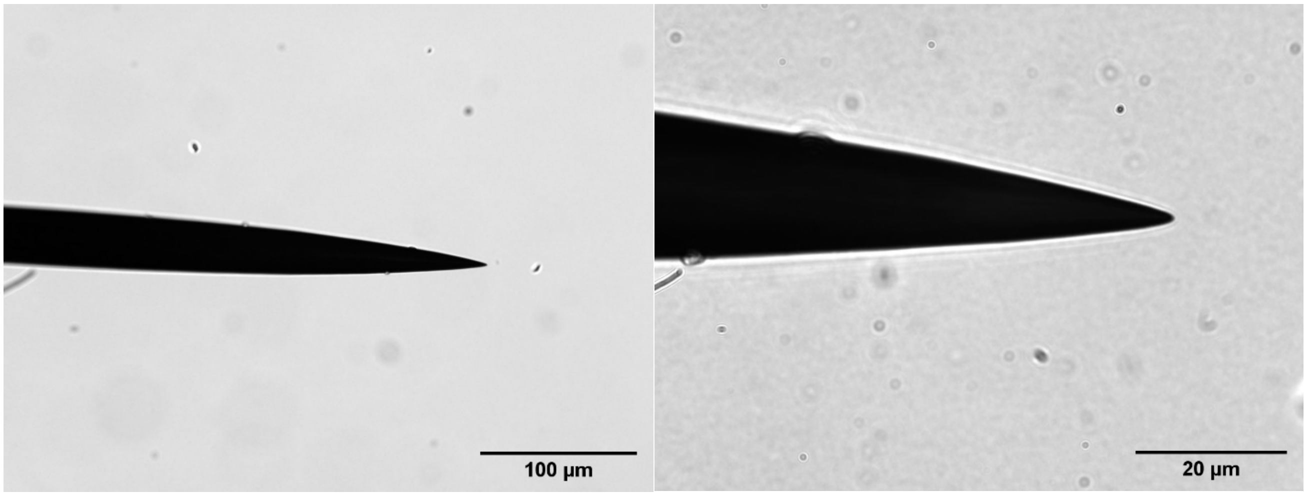

Fig. S2: Tungsten tip used as a microindenter with a tip radius of 1 µm at different magnifications under a light microscope.

Detailed results of the force-indentation measurements

Tab. S1 summarizes all experimental results, in particular mean values ±SD for different mechanical parameters, separated by larval development stages and average values for all stages.

The following parameters are considered: The shell puncture force F_z^{punct}, the maximal effective stiffness at shell rupture $k(F_z^{punct})$, indentation depth at shell puncture Δz^{punct}, embryo elongation Δl and embryo widening Δw upon indentation with a given force F_z (see main text), the shell elastic modulus E_{shell} and the maximal interior pressure at shell rupture p_{int}^{max}.

Untreated	All stages			Early			Mid			Late		
	Mean	SD	n	Mean	SD	n	Mean	SD	n	Mean	SD	n
F_z^{punct} [µN]	48.86	12.78	28	46.53	21.97	5	47.03	6.18	6	50.19	12.19	17
$k(F_z^{punct})$ [N/m]	9.61	2.85	28	8.86	4.18	5	8.09	1.74	6	10.36	2.60	17
Δz^{punct} [µm]	13.85	2.29	28	13.54	4.35	5	13.45	1.40	6	14.09	1.83	17
Δl [µm]	0.35	0.36	29	0.33	0.42	6	0.52	0.46	6	0.30	0.30	17
Δw [µm]	1.11	0.68	29	1.38	1.00	6	0.87	0.43	6	1.10	0.63	17
E_{shell} [Pa]	1.12E+08	2.62E+07	27	1.25E+08	3.16E+07	5	1.20E+08	3.67E+07	6	1.04E+08	1.81E+07	16
p_{int}^{max} [Pa]	1.20E+05	2.16E+04	28	1.12E+05	2.47E+04	5	1.24E+05	2.08E+04	6	1.21E+05	2.18E+04	17
**Bleached 2min**	All stages			Early			Mid			Late		
	Mean	SD	n	Mean	SD	n	Mean	SD	n	Mean	SD	n
F_z^{punct} [µN]	16.92	5.31	20	18.46	2.64	3	16.54	8.62	6	16.7	4.13	11
$k(F_z^{punct})$ [N/m]	3.31	2.04	20	3.69	2.19	3	3.59	2.57	6	3.06	1.87	11
Δz^{punct} [µm]	11.30	3.94	20	10.64	0.76	3	12.70	6.84	5	10.89	2.92	12
Δl [µm]	0.46	0.63	20	0.55	0.21	4	0.40	0.37	5	0.45	0.83	11
Δw [µm]	2.47	0.92	20	1.78	0.85	4	2.26	0.90	5	2.81	0.84	11
E_{shell} [Pa]	8.65E+07	6.15E+07	20	7.50E+07	1.73E+07	3	7.33E+07	5.38E+07	6	9.68E+07	7.36E+07	11
p_{int}^{max} [Pa]	5.02E+04	1.76E+04	19	5.24E+04	9.97E+03	3	4.75E+04	2.30E+04	6	5.11E+04	1.73E+04	10
**Bleached 5min**	All stages			Early			Mid			Late		
	Mean	SD	n	Mean	SD	n	Mean	SD	n	Mean	SD	n
F_z^{punct} [µN]	6.78	2.77	18	5.65	3.07	5	7.25	4.51	3	7.20	2.39	10
$k(F_z^{punct})$ [N/m]	1.11	0.57	18	0.75	0.27	5	1.24	0.40	3	1.24	0.68	10
Δz^{punct} [µm]	10.76	3.60	18	11.16	4.31	5	10.96	2.17	3	10.52	1.11	10
Δl [µm]	0.56	0.74	19	0.73	0.31	6	1.33	0.50	3	0.22	0.16	10
Δw [µm]	2.43	0.90	19	2.23	0.30	6	2.83	0.88	3	2.42	0.27	10
E_{shell} [Pa]	4.67E+07	5.16E+07	18	1.50E+07	1.00E+07	3	3.17E+07	1.53E+07	3	5.10E+07	5.66E+07	10
p_{int}^{max} [Pa]	2.33E+04	1.17E+04	18	1.87E+04	1.28E+04	3	2.29E+04	1.43E+04	3	2.48E+04	1.24E+04	10
**Chitinase**	All stages			Early			Mid			Late		
	Mean	SD	n	Mean	SD	n	Mean	SD	n	Mean	SD	n
F_z^{punct} [µN]	0.54	0.37	21	5.57E-07	3.1E-07	14	3.43E-07	0	1	6.24E-07	5.86E-07	5
$k(F_z^{punct})$ [N/m]	0.05	0.04	20	0.05	0.05	14	0.02	0	1	0.06	0.06	4
Δz^{punct} [µm]	10.22	4.11	20	10.45	4.77	14	6.88	0	1	10.24	1.87	5
Δl [µm]	-0.62	2.39	21	-1.18	2.65	14	-1.20	0	1	0.77	1.13	6
Δw [µm]	5.19	2.44	21	6.31	2.07	14	5.40	0	1	2.53	0.85	6
E_{shell} [Pa]	6.50E+06	4.89E+06	20	7.14E+06	5.79E+06	14	5.00E+06	0.00E+00	1	5.00E+06	0.00E+00	5
p_{int}^{max} [Pa]	3.77E+03	1.72E+03	20	4.06E+03	1.89E+03	14	1.80E+03	0.00E+00	1	3.35E+03	1.00E+03	5

Tab. S1: Experimental results of all force-indentation measurements (mean values ± SD, *n* = number of embryos).
